# Depression and anxiety in Chinese pregnant women in the mid-phase of the COVID-19 pandemic: a cross-sectional study

**DOI:** 10.3389/fgwh.2025.1641022

**Published:** 2025-09-29

**Authors:** Su-Fen Qi, Wei-Hong Zhang, Li-Yan Du, Jie Hu

**Affiliations:** 1 Hebei Key Laboratory of Environment and Human Health, Department of Social Medicine and Health Care Management, School of Public Health, Hebei Medical University, Shijiazhuang, China; 2 International Centre for Reproductive Health, University of Ghent, Ghent, Belgium; 3 Center Information Department, Hebei Center for Women and Children's Health, Shijiazhuang, China; 4 Department of Science and Technology, Hebei Medical University, Shijiazhuang, Hebei, China

**Keywords:** epidemic cognition, depression, anxiety, maternal health services, pregnancy, COVID-19

## Abstract

**Background:**

This study aimed to evaluate the prevalence and associated factors of depressive and anxiety symptoms among Chinese pregnant women during the middle period of COVID-19.

**Methods:**

From May to August 2021, a cross-sectional online survey was conducted among pregnant women in Shijiazhuang, Hebei Province. The data collected included demographic characteristics (age, occupation, region, parity, number of fetuses, pregnancy-related disorders, education level, awareness of common symptoms, attention to the epidemic, and frequency of temperature measurement). We recruited participants using a convenience sampling approach. Depression and anxiety were assessed using self-depression rating scale (SDS) and a self-rating anxiety scale (SAS). A univariate and multivariable binomial logistic regression model was applied to identify risk factors for depression and anxiety.

**Results:**

Cronbach's α coefficients for SDS and SAS were 0.837 and 0.826, respectively. Among 1,036 participants, the prevalence of depressive and anxiety symptoms was 59.8% (620 cases) and 6.7% (69 cases), respectively. Factors associated with depression included the number of fetuses (OR = 2.98, 95% CI 1.22–7.31), education level (OR = 0.58, 95% CI 0.45–0.75), attention to the epidemic (OR = 0.65, 95% CI 0.42–0.91), and frequency of temperature measurement (OR = 0.62, 95% CI 0.41–0.93). Factors associated with anxiety included parity (OR = 0.51, 95% CI 0.31–0.83), attention to the epidemic (OR = 2.14, 95% CI 1.18–3.89), and frequency of temperature measurement (OR = 2.86, 95% CI 1.08–7.52). Multivariate binomial logistic regression analysis indicated that a higher education level was an associated factor for depression (adjusted OR = 0.52, 95% CI 0.38–0.70). However, the parity (adjusted OR = 0.46, 95% CI 0.26–0.82) and pregnancy-related disorders (adjusted OR = 2.55, 95% CI 1.46–4.45) were independent associated factors for anxiety.

**Conclusion:**

Pregnant women with lower education levels, primipara status, and pregnancy-related disorders were association with higher levels of depression and anxiety during the middle period of COVID-19. These findings suggest the need for targeted interventions to support the mental health of pregnant women during pandemics.

## 1 Introduction

Maternal mental health is a significant public health concern, given its short- and long-term effects on both women and children's health (1, 2). Research indicates that untreated symptoms of depression and anxiety can lead to numerous complications during pregnancy and affect newborns (e.g., spontaneous abortion, weakened immunity, operative delivery, cesarean section, preterm birth, and lower birthweight), as well as influencing children's health outcomes (e.g., reduced immunity, impaired cognitive development, and behavioral and emotional difficulties) and mother-infant bonding (risk factors for bonding difficulties) (1–3).

Since it was first identified in December 2019, the 2019 novel coronavirus (COVID-19) has rapidly spread across the globe (4). Each province in China continuously adjusted its pandemic control strategies in response to the local epidemiological situation. From January to April 2021, a large-scale outbreak occurred in Shijiazhuang, Hebei province, leading to a complete lockdown of the city along with strict measures to contain the virus. These measures included the suspension of inter-city transportation and intra-city public transit, restricting movement outside communities, and encouraging residents to remain at home. The physical and social repercussions of the pandemic are potentially devastating. The health implications are serious, involving fatalities, overwhelmed healthcare systems, and economic instability. Pregnancy is a particularly vulnerable period during which psychological distress can adversely affect both the mother and the baby.

Women tend to report higher symptoms of depression and anxiety during disease outbreaks compared to men (5–7). Pregnant women are particularly vulnerable to the impacts of the COVID-19 crisis, highlighting the urgent need for measures to protect this population (8). During the pandemic, pregnant women experienced significant challenges in accessing essential healthcare services (9), citing concerns over COVID-19 exposure, childcare, breastfeeding, and vaccination (10), all of which have further affected their psychological well-being. Since the onset of the COVID-19 pandemic, women in the perinatal period have been identified as a vulnerable group due to the potential impact of alterations in their immune systems, which may predispose them to more severe respiratory symptoms from COVID-19 infection (11). More importantly, women who contracted COVID-19 during pregnancy face an increased risk of preterm delivery, maternal mortality, and neonatal death (12). However, the likelihood of vertical transmission of the virus remains relatively low (7).

The COVID-19 pandemic has significantly affected the mental health and psychological functioning of the global population, exacerbating the prevalence of depression and other common mental disorders. Previous studies have examined the prevalence of prenatal depression and anxiety symptoms at various stages and across different countries (13–20); however, our understanding of these issues among pregnant women in China is still limited. An umbrella review and meta-analytic synthesis indicated that the global prevalence of antenatal and postpartum depression was 29% and 26%, respectively. For anxiety, the pooled prevalence for antenatal and postnatal cases during the COVID-19 pandemic was reported at 31% (17). Notably, a large variability in prevalence rates of perinatal depression and anxiety was observed in these studies, indicating a generally high level of heterogeneity (21). Several studies have explored the factors contributing to mental health issues in pregnant women during the pandemic (17). Commonly identified factors include social isolation, economic stress, and concerns about infection (22, 23). However, these studies have primarily focused on specific regions or populations, limiting the generalizability of their findings. Thus, this study aims to evaluate the prevalence and relevant factors associated with depressive and anxiety symptoms among pregnant women in Shijiazhuang City during the midpoint of the COVID-19 pandemic. We hypothesize that factors such as lower education levels, primipara, and pregnancy-related disorders may have a significant impact on the prevalence of depression and anxiety symptoms during the pandemic. By employing a comprehensive and multi-faceted approach, our study provides a broader understanding of the mental health challenges faced by pregnant women during this unprecedented time.

## 2 Methods and materials

This descriptive study adheres to the STROBE Statement, ensuring rigorous and transparent reporting of our cross-sectional investigation.

### 2.1 Study population

This study was conducted from May to August 2021, involving pregnant women residing in Shijiazhuang City. Pregnancy status was confirmed via B-ultrasonography, and participants were required to complete an online psychological assessment questionnaire at their first prenatal care visit. Inclusion criteria comprised: (1) Women who permanently reside in Shijiazhuang District; (2) Pregnant women who consented to complete the survey. Exclusion criteria included: (1) Inability to use a mobile phone to scan the code to access the questionnaire; (2) History of severe mental illness prior to pregnancy.

### 2.2 Data collection

In this study, we recruited participants using a convenience sampling approach. Pregnancy status was confirmed via B-ultrasonography, and participants were required to complete an online psychological assessment questionnaire at their first prenatal care visit. To address the representativeness of the sample, we collected as many questionnaires as possible to provide greater statistical power. We employed various recruitment channels. These included online platforms, social media, community health centers, and healthcare facilities. We also utilized existing networks and partnerships with local organizations to expand our reach.

Two approaches were taken to avoid response biases: (1) Use neutral and clear questions: Avoid leading, double-barreled, or loaded questions that may influence responses. (2) Avoid using jargon: Use clear and straightforward language so all respondents interpret questions the same way. We designed the survey to be concise and straightforward to reduce respondent fatigue and increase response rates. Four approaches were taken to avoid no response: (1) The survey was designed to be completed in less than 10 min to minimize participant burden. (2) To maximize response rates, we implemented a system of multiple reminders. Participants who did not respond to the initial invitation received follow-up reminders via messaging platforms, such as WeChat groups, to encourage their participation. (3) In the introduction part of the survey, we clearly stated the focus and purpose of the study. Participants were informed about the importance of their contribution and how the results would be used. (4) We ensured that the questions were easy to respond to by using pre-selection options where appropriate. This made it quicker and easier for participants to complete the survey. Specifically, we distributed questionnaires through “Wenjuanxing” software which is very frequent use and Populaire in Chinese survey. Participants were informed of the purpose of the survey and decided on their own whether to complete the questionnaire. The advantage of this sampling method is that it allows for rapid data collection, and participants usually have a higher level of interest and cooperation regarding the survey topic.

The questionnaire used was designed by researchers. The researchers entered the finalized questionnaire into the “Wenjuanxing” software, generating a two-dimensional code or website link that allowed eligible pregnant women to complete the survey by scanning the code with their mobile devices.

### 2.3 Questionnaire content

The questionnaire included demographic characteristics, including age, occupation, region, parity, number of fetuses, pregnancy-related disorders, education level, awareness of common symptoms, levels of attention to the epidemic, and frequency of temperature measurement. Additionally, it assessed symptoms of depression and anxiety using self-depression rating scale (SDS) and a self-rating anxiety scale (SAS).

#### 2.3.1 Independent variables

Participants' basic demographic characteristics included age (18–24, 25–34, or ≥35), occupation (employed vs. unemployed), region (urban, suburban, or rural), parity (primipara vs. multipara), number of fetuses (single, twins, or triplets and above), pregnancy-related disorders (present vs. absent), education level (high school and below, university degree, or postgraduate), awareness of common symptoms (know, somewhat know, or do not know), levels of attention to the epidemic (multiple times a day, once a day, or once every few days), and frequency of temperature measurement (multiple times a day, once a day, or every few days).

#### 2.3.2 Evaluation of depressive symptoms and anxiety symptoms

Pregnant volunteers were requested to complete the SAS (24) and SDS (25), reflecting their recent emotional experiences. Both the SAS and SDS are widely utilized tools for evaluating an individual's mental state and were developed by Zung et al., receiving recommendations from the United States Department of Education, Health, and Welfare (26, 27). Each scale comprises 20 questions addressing psychotic emotional symptoms, psychomotor disturbances, somatic disorders, and mental health issues related to anxiety or depression. Participants selected the response that best represented their mental state for each question. Responses were scored using a Likert-type scale ranging from 1–4, or through reverse scoring (options included “a little of the time,” “some of the time,” “a good part of the time,” and “most of the time”). The raw total scores, derived from the cumulative scores of the 20 questions, were subsequently converted into percentile standard scores. A standard score of 50 on the SAS was established as the threshold for identifying anxiety symptoms, whereas a score of 53 on the SDS served as the threshold for depression symptoms.

### 2.4 Sample size calculation

A previous study indicated that the prevalence of depression and anxiety among Chinese women during pregnancy was 5.2% and 8.0%, respectively (28, 29). Within the context of the COVID-19 pandemic, a 10% increase in these prevalence rates was deemed significant for assessing the likelihood of anxiety or depression during pregnancy. The sample size was calculated using the standard formula $n=Z_{\alpha /2}^{2}P(1-P)/\delta^{2}$, with $Z_{\alpha /2}=1.96$, expected prevalence rates of 15.2% for depression and 18.0% for anxiety, and an allowable error (δ) of 5%. This calculation yielded recommended sample sizes of 199 for depression (*n* = 199) and 227 for anxiety (*n* = 227), with the larger sample size (227) adopted for this study. Considering an anticipated 10% invalid response rate, a total of 252 questionnaires were collected from pregnant women in Shijiazhuang City. The sample calculation was just for having the statistically significant, however, it was good things or added value that we got more participants than we expected using the same resource. It is remarkable that, with all necessary resources already in place, incorporating additional participants into our sample collection process did not incur significant additional costs or burdens. Moreover, from an ethical perspective, ensuring a sufficient sample size enhances the generalizability of our study findings, which is of benefit to the broader scientific community. Therefore, there were 1,242 electronic questionnaires completed during the study period.

### 2.5 Quality control

In adherence to principles of scientific rigor and feasibility, the researchers initiated the questionnaire design phase by reviewing a substantial body of relevant literature. They then defined the content of the questionnaire through expert consultations and group discussions. Following this, the questionnaire underwent modifications and refinements based on a preliminary survey to ensure its usability. Before participants filled out the electronic questionnaire via a scan code, the researchers communicated the study's purpose, emphasizing the principle of voluntary participation for pregnant women.

All entries were designated as compulsory questions, and the IP address verified through the mobile phone of the tester could only retain the final answer provided on the test day. Questionnaires were submitted only upon the completion of all items; otherwise, the system would automatically classify the submission as incomplete.

To mitigate potential response biases, we designed the survey to be concise and straightforward to reduce respondent fatigue and increase response rates. The survey was designed to be completed in less than 10 min to minimize participant burden. To further improve the response rate, we made multiple attempts to contact potential respondents to maximize participation. This strategy helps in reaching a broader audience and reduces the chances of non-response bias by ensuring that individuals have multiple opportunities to respond.

### 2.6 Statistics analysis

All raw data obtained from the “Wenjuanxing” software were imported into Excel 2016, where a preliminary database was established following coding and sorting. Reliability refers to the consistency and stability of the measurement results obtained from a questionnaire, and we used the method of “Internal Consistency Reliability”. Cronbach's α coefficient was utilized to assess the internal consistency reliability of the SDS and the SAS. An alpha value of 0.70 or higher is typically considered to indicate good internal consistency. The distribution of categorical data is described using frequencies (percentages), which include demographic characteristics, the SDS, and the SAS. The Pearson chi-square test was employed to analyze the differences in detection rates of depressive and anxiety symptoms across demographic characteristics. A univariate and multivariate binomial logistic regression model was applied to evaluate potential risk factors for depression and anxiety, with the odds ratio (OR) and its 95% confidence interval (CI) calculated. All statistical tests conducted in this study were two-tailed, using an alpha level of 0.05. All statistical analyses were performed using SPSS 23.0 software.

### 2.7 Ethics approval and consent to participate

The study was approved by the Ethics Committee of Hebei Medical University (No: 2021116). All participants provided their consent to take part in this study and signed an informed consent form.

## 3 Results

**Figure 1:**
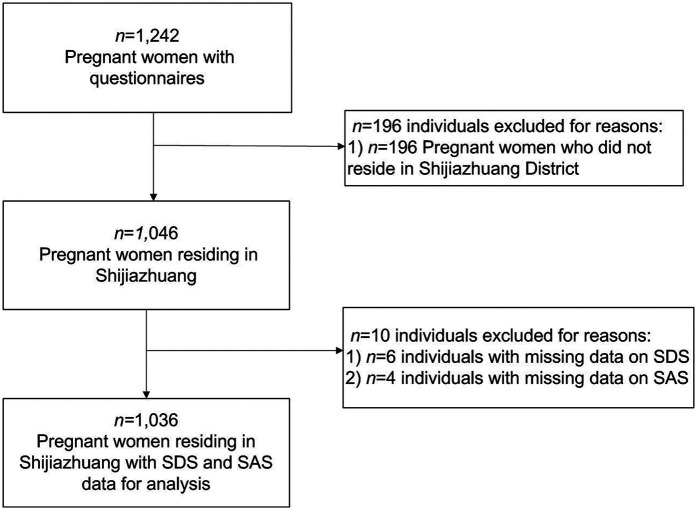
Flow chart illustrating the sample selection for the present study.

Out of 1,242 electronic questionnaires completed during the study period, 1,036 (83.41%) were included in the final analysis after removing invalid responses (see Figure 1).

### 3.1 Demographic characteristics of the subjects

The demographic characteristics of the 1,036 pregnant women are presented in Table 1. In terms of age distribution, most women (73.3%) were aged 25–34 years, whereas 12.8% were aged 18–24 years. Regarding occupational distribution, 366 women (35.3%) were employed, and over half (59.5%) of the participants resided in rural areas. Most of the women (63.0%) were multiparas. Pregnant women with single fetuses comprised 93.1% of the sample, whereas those experiencing pregnancy complications accounted for 16.2%. In relation to educational attainment, the largest proportion of participants had completed high school or lower (58.9%), with only a small percentage (1.4%) possessing postgraduate degrees.

**Table 1:** Demographic characteristics of the study subjects.

Variable	*n* (%)
Age (years)	
18–24	133 (12.8)
25–34	759 (73.3)
≥35	144 (13.9)
Occupation	
Employed	366 (35.3)
Unemployed	670 (64.7)
Region	
Urban	229 (22.1)
Suburban	191 (18.4)
Rural	616 (59.5)
Parity	
Primipara	383 (37.0)
Multipara	653 (63.0)
Number of fetuses	
Single	964 (93.1)
Twins	40 (3.9)
Triplets and above	32 (3.1)
Pregnancy-related Disorders	
No	868 (83.8)
Yes	168 (16.2)
Education	
High school and below	610 (58.9)
University degree	411 (39.7)
Postgraduate	15 (1.4)
Awareness of common symptoms	
Know	981 (94.7)
Somewhat Know	48 (4.6)
Don't know	7 (0.7)
Levels of attention to the epidemic	
Multiple times a day	354 (34.2)
Once a day	455 (43.9)
Once every few days	227 (21.9)
Frequency of temperature measure	
Multiple times a day	143 (13.8)
Once a day	573 (55.3)
every few days	320 (30.9)

As shown in Table 1, 981 participants (94.7%) were aware of common symptoms, whereas only 7 (0.7%) were unaware. In terms of attention to the epidemic, 455 (43.9%) pregnant women checked the news daily, whereas only 227 (21.9%) paid attention every few days. Regarding the frequency of temperature measurement, most pregnant women (55.3%) measured their temperature once a day, whereas only 13.8% measured it multiple times daily.

### 3.2 Depression and anxiety symptoms

#### 3.2.1 Detection rate of depression and anxiety symptoms in pregnant women during the epidemic of COVID-19

Cronbach's α coefficients for SDS and SAS were 0.837 and 0.826, respectively. The above results indicate that the measurement results of our questionnaire are consistent and stable.

As shown in Table 2, among 1,036 participating pregnant women, the prevalence of depressive and anxiety symptoms was 620 (59.8%) and 69 (6.7%), respectively. Univariate logistics regression analysis of depression showed that there were no statistically significant differences in depression among pregnant women of different ages, occupations, regions, parity, pregnancy disorders, and awareness of common symptoms (all *P* > 0.05).The factors associated with depression included the number of fetuses (OR = 2.98, 95% CI 1.22–7.31), educational level (OR = 0.58, 95% CI 0.45–0.75), level of attention to the epidemic (OR = 0.65, 95% CI 0.42–0.91), and frequency of temperature measurement (OR = 0.62, 95% CI 0.41–0.93). There were significant differences among pregnant women with different numbers of fetuses (*P* = 0.042); as the number of fetuses increased, depression levels increased. Significant differences were observed among pregnant women with different educational levels (*P* < 0.001); as educational level increased, depression rates decreased. There were significant differences among pregnant women with varying levels of attention to the epidemic (*P* = 0.033); as attention to the epidemic increased, depression rates increased. Significant differences were also found based on the frequency of temperature measurement (*P* = 0.016); higher measurement frequency was associated with increased depression rates. Univariate logistics regression analysis of anxiety showed that there were no statistically significant differences in anxiety among pregnant women of different ages, occupations, regions, number of fetuses, education levels, or awareness of common symptoms (all *P* > 0.05). Factors associated with anxiety included parity (OR = 0.51, 95% CI 0.31–0.83), levels of attention to the epidemic (OR = 2.14, 95% CI 1.18–3.89), and frequency of temperature measurement (OR = 2.86, 95% CI 1.08–7.52). Statistically significant differences were observed for parity (*P* = 0.008); primiparas had higher anxiety levels compared with multiparas. Significant differences were also found among pregnant women with and without pregnancy disorders (*P* < 0.001); those with pregnancy disorders had higher anxiety levels (13.7%) compared to those without (5.3%). Differences were also noted based on levels of epidemic awareness (*P* = 0.002); higher awareness was associated with increased anxiety prevalence. Finally, differences were observed regarding temperature measurement frequency (*P* = 0.042); higher frequency was associated with lower anxiety prevalence.

**Table 2:** Univariate logistics regression analysis of depression and anxiety symptoms in pregnant women during the COVID-19 epidemic.

Variable	Depression	*P*	Crude OR (95% CI)	Anxiety	*P*	Crude OR (95% CI)
All participants	620 (59.8)			69 (6.7)		
Age (years)		0.726			0.265	
18–24	79 (59.4)		1.00 (Reference)	9 (6.8)		1.00 (Reference)
25–34	459 (60.0)	0.815	1.05 (0.72–1.52)	55 (7.2)	0.843	1.08 (0.52–2.23)
≥35	82 (56.9)	0.679	0.90 (0.56–1.46)	5 (3.5)	0.219	0.50 (0.16–1.52)
Occupation		0.504			0.720	
Employed	214 (58.5)		1.00 (Reference)	23 (6.3)		1.00 (Reference)
Unemployed	406 (60.6)	0.504	1.09 (0.84–1.42)	46 (6.9)		1.10 (0.66–1.85)
Region		0.432			0.712	
Urban	133 (58.1)		1.00 (Reference)	18 (7.9)		1.00 (Reference)
Suburban	122 (63.9)	0.226	1.23 (0.86–1.90)	12 (6.3)	0.533	0.79 (0.37–1.68)
Rural	365 (59.3)	0.758	1.05 (0.77–1.43)	39 (6.3)	0.432	0.79 (0.44–1.42)
Parity		0.180			0.008	
Primipara	219 (57.2)		1.00 (Reference)	36 (9.4)		1.00 (Reference)
Multipara	401 (61.4)		1.19 (0.92–1.54)	33 (5.1)		0.51 (0.31–0.83)
Number of fetuses		0.042			0.973	
Single	571 (59.2)		1.00 (Reference)	64 (6.6)		1.00 (Reference)
Twins	23 (57.5)	0.827	0.93 (0.49–1.77)	3 (7.5)	0.831	1.14 (0.34–3.80)
Triplets and above	26 (81.3)	0.017	2.98 (1.22–7.31)	2 (6.3)	0.931	0.94 (0.22–4.01)
Pregnancy-related Disorders		0.261			<0.001	
No	526 (60.6)		1.00 (Reference)	46 (5.3)		1.00 (Reference)
Yes	94 (56.0)		0.82 (0.59–1.15)	23 (13.7)		2.83 (1.67–4.82)
Education		<0.001			0.122	
High school and below	398 (65.2)		1.00 (Reference)	37 (6.1)		1.00 (Reference)
University degree	214 (52.1)	<0.001	0.58 (0.45–0.75)	29 (7.1)	0.528	1.18 (0.71–1.94)
Postgraduate	8 (53.3)	0.344	0.61 (0.22–1.70)	3 (20.0)	0.043	3.87 (1.05–14.3)
Awareness of common symptoms		0.920			0.274	
Know	586 (59.7)		1.00 (Reference)	63 (6.4)		1.00 (Reference)
Somewhat Know	30 (62.5)	0.703	1.12 (0.68–2.04)	6 (12.5)	0.107	2.08 (0.85–5.08)
Don't know	4 (57.1)	0.889	0.90 (0.20–4.04)	0 (0)	0.999	0.00 (0.00–0.00)
Levels of attention to the epidemic		0.033			0.002	
Multiple times a day	230 (65.0)		1.00 (Reference)	21 (5.9)		1.00 (Reference)
Once a day	266 (58.5)	0.060	0.76 (0.57–1.01)	21 (4.6)	0.403	0.77 (0.41–1.43)
Once every few days	124 (54.6)	0.013	0.65 (0.42–0.91)	27 (11.9)	0.012	2.14 (1.18–3.89)
Frequency of temperature measure		0.016			0.042	
Multiple times a day	93 (65.0)		1.00 (Reference)	5 (3.5)		1.00 (Reference)
Once a day	356 (62.1)	0.520	0.88 (0.60–1.29)	34 (5.9)	0.256	1.74 (0.67–4.53)
Every few days	171(53.4)	0.020	0.62 (0.41–0.93)	30 (9.4)	0.034	2.86 (1.08–7.52)

#### 3.2.2 Multivariable analysis factors affecting depression and anxiety

Multivariable logistic regression with backward stepwise selection was used to determine the factors associated with depression and anxiety in pregnant women. The dependent variable in our model was a binary indicator of the presence (1) or absence (0) of depression and anxiety symptoms, as assessed by standardized questionnaires (e.g., SDS and SAS). The clinically/theoretically important demographic characteristics of the participants, such as age, occupation, region, parity, number of fetuses, pregnancy-related disorders, education level, awareness of common symptoms, levels of attention to the epidemic, and frequency of temperature measurement, served as potential confounders and covariates in the study. The specific assignment values are detailed in Supplementary eTable S1. Hosmer–Lemeshow goodness-of-fit test: SAS (*χ*² = 8.087, df = 8, *P* = 0.432) and SDS (*χ*² = 9.421, df = 8, *P* = 0.308). These results confirm adequate fit and absence of concerning collinearity. The adjusted OR and its 95% CIs were calculated to analyze the association between various characteristics and the presence of depression/anxiety symptoms. As shown in Table 3, the multivariable binary logistic regression analysis indicated that a higher education level served as an associated factor against depression (adjusted OR = 0.52, 95% CI 0.38–0.70). This suggests that education level significantly influences depression in pregnant women, with an adjusted OR of 0.52 indicating that increased education corresponds to a decreased risk of depression.

**Table 3:** Multivariate binomial logistic regression analysis of depression and anxiety symptoms in pregnant women during the COVID-19 epidemic.

Variable	Depression		Anxiety	
	*P*	Adjusted OR (95% CI)	*P*	Adjusted OR (95% CI)
Age (years)	0.726		0.287	
18–24		1.00 (Reference)		1.00 (Reference)
25–34	0.893	0.97 (0.64–1.47)	0.206	1.68 (0.75–3.75)
≥35	0.164	0.68 (0.4–1.17)	0.988	1.01 (0.29–3.48)
Occupation	0.576		0.501	
Employed		1.00 (Reference)		1.00 (Reference)
Unemployed		0.92 (0.68–1.24)		1.23 (0.67–2.28)
Region	0.196		0.831	
Urban		1.00 (Reference)		1.00 (Reference)
Suburban	0.342	1.22 (0.81–1.84)	0.838	0.83 (0.38–1.83)
Rural	0.473	0.88 (0.63–1.24)	0.465	0.84 (0.44–1.61)
Parity	0.976		0.009	
Primipara		1.00 (Reference)		1.00 (Reference)
Multipara		1 (0.75–1.35)		0.46 (0.26–0.82)
Number of fetuses	0.098		0.716	
Single		1.00 (Reference)		1.00 (Reference)
Twins	0.960	0.98 (0.51–1.9)	0.544	1.48 (0.42–5.19)
Triplets and above	0.032	2.74 (1.09–6.89)	0.558	1.57 (0.35–7.06)
Pregnancy-related Disorders	0.554		0.001	
No		1.00 (Reference)		1.00 (Reference)
Yes		0.90 (0.64–1.28)		2.55 (1.46–4.45)
Education	<0.001		0.110	
High school and below		1.00 (Reference)		1.00 (Reference)
University degree	<0.001	0.52 (0.38–0.70)	0.110	0.89 (0.49–1.62)
Postgraduate	0.273	0.55 (0.19–1.60)	0.712	3.90 (0.94–16.18)
Awareness of common symptoms	0.780		0.646	
Know		1.00 (Reference)		1.00 (Reference)
Somewhat Know	0.489	1.25 (0.66–2.35)	0.350	1.58 (0.6–4.15)
Don't know	0.926	0.93 (0.20–4.31)	0.999	0 (0–0)
Levels of attention to the epidemic	0.128		0.030	
Multiple times a day		1.00 (Reference)		1.00 (Reference)
Once a day	0.061	0.75 (0.55–1.01)	0.183	0.64 (0.34–1.23)
Once every few days	0.108	0.72 (0.49–1.07)	0.202	1.57 (0.78–3.16)
Frequency of temperature measure	0.152		0.397	
Multiple times a day		1.00 (Reference)		1.00 (Reference)
Once a day	0.900	0.97 (0.65–1.46)	0.201	1.92 (0.71–5.2)
every few days	0.163	0.72 (0.46–1.14)	0.186	2.04 (0.71–5.85)

A similar approach was adopted to assess the effects of parity, pregnancy disorders, attention to the epidemic, and frequency of temperature measurement on anxiety. As shown in Table 3, The results demonstrated that parity (adjusted OR = 0.46, 95% CI 0.26–0.82) and the presence of pregnancy disorders (adjusted OR = 2.55, 95% CI 1.46–4.45) were significant factors influencing anxiety. Specifically, the adjusted OR for parity of 0.46 suggests that pregnant women with a history of childbirth tend to experience lower levels of anxiety, indicating that such a history acts as an associated factor. Conversely, the adjusted OR for pregnancy disorders of 2.55 highlights that pregnant women experiencing these disorders face a higher risk of anxiety, thereby categorizing pregnancy disorders as an associated factor.

## 4 Discussion

Among the 1,036 participating pregnant women, the prevalence of depression and anxiety was found to be 620 (59.8%) and 69 (6.7%), respectively. The results of the binary logistic regression analysis indicated that education level is a significant influencing factor for depression among pregnant women, serving as an associated factor. Additionally, the findings revealed that parity and the presence or absence of pregnancy disorders are important factors influencing anxiety in this population.

Anxiety and depression are prevalent among pregnant women, with detection rates differing across various countries and regions. Generally, it is observed that approximately 4%–15% of pregnant women experience symptoms of depression, whereas 5%–13% report symptoms of anxiety. Furthermore, the co-occurrence of both depression and anxiety occurs in 0.9%–3.8% of this population (30). In our study, we found that during the peak of the COVID-19 pandemic, the prevalence of depression among pregnant women in Shijiazhuang was notably high at 59.8%. In contrast, the proportion of those experiencing anxiety symptoms was much lower at 6.7%. These findings are consistent with reports on the mental health status of pregnant women in China during the pandemic, which indicated that 8.3% of patients experienced anxiety, whereas 50.6% showed signs of depression (31). This data suggests that whereas the level of anxiety among pregnant women has remained stable compared to pre-pandemic times, the prevalence of depression has significantly increased. The pandemic appears to have a more pronounced effect on the mental health of individuals, especially in terms of contributing to depressive symptoms (32). Moreover, past research indicates that the implications of depression during pregnancy can be more severe than those associated with anxiety. Depression not only correlates with premature birth (33) but also elevates the risk of low birth weight (34). From an etiological perspective, prenatal depression has a more substantial effect on low birth weight than it does on preterm birth (35). Therefore, it is crucial to prioritize the mental health of pregnant women, especially during the COVID-19 pandemic, with a particular focus on addressing depression. Timely psychological counseling should be made readily available to this vulnerable population.

The prevalence of depression is remarkably high at approximately 60%, while the prevalence of anxiety is extremely low at 6.7%, creating a striking disparity. Given that both conditions are often reported to be correlated to some degree in the context of mental state during pregnancy, it is essential to provide a comprehensive interpretation and discussion of the reasons for such a large discrepancy. This discrepancy is likely due to a combination of biological, psychosocial, measurement, and cultural factors. (1) Biological Factors: Hormonal fluctuations during pregnancy, particularly changes in estrogen and progesterone, significantly impact emotional regulation and are more strongly linked to depression than anxiety. Additionally, neurotransmitter imbalances, such as those involving serotonin and dopamine, are associated with both depression and anxiety, but pregnancy-related changes may disproportionately affect depression (36). (2) Measurement Tools and Diagnostic Criteria: The SDS is more sensitive to depression, while the SAS is conservative for anxiety, potentially overestimating depression and underestimating anxiety prevalence (36, 37). (3) Cultural and Societal Factors: Cultural contexts and societal focus may lead to higher reporting rates of depression compared to anxiety, as depression symptoms are more readily recognized and reported, while anxiety is often overlooked or underreported (36). (4) Psychosocial Factors: These factors include sociopsychological elements such as stress and social support (38). Prior research has indicated that anxiety is associated with heightened engagement in threat-avoidance behaviors, whereas depression is linked to diminished participation in reward-seeking behaviors (35, 39). When the questionnaires were distributed, the epidemic in Shijiazhuang had reached a stable phase, occurring more than three months after the city was temporarily closed (from January to April 2021). At that point, the number of new COVID-19 cases in Shijiazhuang was gradually declining, and the number of patients recovering was on the rise. News coverage during the epidemic highlighted China's significant efforts and commitment to controlling COVID-19, including reports on the successful deliveries of pregnant women diagnosed with the virus. Additionally, during the outbreak, family members of pregnant women were at home, potentially increasing the time spent with them and strengthening social support. Consequently, we hypothesized that transparent communication, a stable epidemic situation, and enhanced social support may have mitigated the threat-avoidance behaviors of pregnant women with COVID-19, which could explain why their anxiety levels did not escalate significantly during the epidemic. In contrast, the “shelter in place” measures required pregnant women to remain at home or in isolation during the outbreak, preventing them from undergoing obstetric examinations or engaging in various social activities. These challenges may have diminished their participation in reward-seeking behaviors, leading to a pronounced experience of depression among pregnant women during the epidemic. A cross-sectional study design may not accurately reflect the dynamic changes in depression and anxiety during pregnancy. For instance, depression may be more common during certain stages of pregnancy, while anxiety may be more prevalent at other times (38).

The factors influencing the mental state of pregnant women are complex and multifaceted, including age, education level, occupation, gestational week, economic status, medical history, and sociopsychological components. It is essential to explore potential associated factors to guide preventive measures in the event of future public health crises. The literature presents conflicting results concerning associated factors, with no consensus reached regarding various sociodemographic and obstetric elements (22, 40–44). Furthermore, multiple studies have not shown a significant association between sociodemographic variables and elevated levels of anxiety or depression (31, 40, 44). Consistently, our data did not reveal any significant associations with age, occupation, current residence, or general awareness of common symptoms.

Results of the binomial logistic regression analysis indicated that education level serves as an associated factor against depression. Additionally, pregnant women with a history of childbirth reported lower anxiety levels, whereas those with gestational diseases experienced heightened anxiety. Our findings revealed that women with higher educational attainment exhibited lower levels of depression, consistent with previous studies (45, 46). This correlation can be explained in two ways. First, higher education is often associated with increased family income, reducing financial concerns related to pregnancy, delivery, and child-rearing. Second, women with advanced education tend to have a better understanding of the processes of pregnancy, childbirth, and child development, allowing them to respond more effectively to emergencies. Cognitive coping strategies, health literacy, or help-seeking behavior also could explain why more highly educated pregnant women may experience lower depression risk through adaptive coping styles and better recognition of—and help-seeking for—mental health symptoms (47). Moreover, our results demonstrated that pregnant women with a history of maternal and childbirth experiences exhibited lower anxiety levels, whereas those facing gestational diseases reported higher anxiety levels. This aligns with findings from prior studies indicating that women who are nulliparous or experiencing high-risk pregnancies are more susceptible to anxiety and depression (45, 48). Nulliparity emerged as a risk factor for increased prenatal distress, corroborating earlier research (49–51).

This study has certain limitations. Firstly, the cross-sectional design precludes establishing causality because it cannot clarify whether the identified factors precede or follow depression/anxiety symptoms. Future research should employ longitudinal or experimental designs to explore these relationships further. Additionally, while we controlled for several potential confounders, residual confounding may still be present. Secondly, the selection of research subjects did not adequately adhere to the principle of randomization, thus failing to achieve complete randomness. The convenience sampling approach may lead to selection bias, as individuals who choose to participate may share certain characteristics (such as a higher level of health awareness or greater interest in the survey topic) that differentiate them from the general population. Also, the data collected by the institute were derived from an online electronic questionnaire and were entirely self-reported by the pregnant women, as participants may have self-selected based on their motivation and interest in pregnancy and mental health topics, which introduces a degree of subjectivity and recall bias. We acknowledge that individuals with limited or no access to the internet may be underrepresented in our sample. Future studies should consider alternative methods to reach those without internet access, such as in-person surveys or phone interviews. Third, although we utilized a questionnaire that has been widely validated in numerous similar studies (52–55), we did not do a validity experiment on the relevant population. Therefore, we also recognize that not conducting independent validity analysis in our current study is a significant limitation and suggest that future studies should conduct independent validity analysis when using this questionnaire to further verify its applicability in different populations. Lastly, the timing of the data collection corresponds to the phase of normalized epidemic prevention and control during the COVID-19 pandemic, which may limit the short-term relevance and applicability of our findings.

## 5 Conclusion

Pregnant women with lower educational levels, primipara status, and pregnancy-related disorders were association with higher levels of depression and anxiety during the middle phase of the COVID-19 pandemic. This highlights the necessity for targeted mental health interventions for pregnant women during this challenging period. Establishing psychological health screening systems within hospitals, communities, and families, tailored to relevant factors, is essential to enhance early prevention efforts. Policymakers must prioritize the mental well-being of pregnant women, whereas families should cultivate greater awareness to facilitate the early identification of mental health issues. Developing mental health prevention programs for expectant mothers is an important public health objective.

## Data Availability

The raw data supporting the conclusions of this article will be made available by the authors, without undue reservation.
