# Supplementary material for: Depression and anxiety in Chinese pregnant women in the mid-phase of the COVID-19 pandemic: a cross-sectional study
Source: Front Glob Womens Health. 2025 Sep 29;6:1641022. doi: 10.3389/fgwh.2025.1641022 (PMC12515816; doi:10.3389/fgwh.2025.1641022)
Supplement: Supplementary file 1 [file Table1.pdf]

eTable 1 The specific assignment values in binomial logistic regression analysis of depression and anxiety symptoms

Variable	Category	Assignment values
Age (years)	18-24	1
	25-34	2
	≥35	3
Occupation	Employed	0
	Unemployed	1
Region	Urban	1
	Suburban	2
	Rural	3
Parity	Primipara	0
	Multipara	1
Number of fetuses	Single	1
	Twins	2
	Triplets and above	3
Pregnancy-related Disorders	No	0
	Yes	1
Education	High school and below	1
	University degree	2
	postgraduate	3
Awareness of common symptoms	Know	1
	Somewhat Know	2
	Don’t know	3
Levels of attention to the epidemic	Multiple times a day	1
	Once a day	2
	Once every few days	3
Frequency of temperature measure	Multiple times a day	1
	Once a day	2
	every few days	3
